# Comments on ‘Impact of the COVID-19 pandemic on alcohol-based hand rub consumption and hand hygiene compliance: a cross-sectional study using digital direct-observation tools in Slovakia’

**DOI:** 10.1016/j.infpip.2026.100520

**Published:** 2026-02-19

**Authors:** S.N. Katkuri, V. Vadhithala, A. Kumar, S. Verma, D. Dedeepya

**Affiliations:** a Department of Community Medicine, Malla Reddy Instiute of Medical Sciences, Malla Reddy Vishwavidyapeeth, Suraram, Hyderabad, Telangana, India; b Dr. D. Y. Patil Medical College, Hospital and Research Centre, Dr. D. Y. Patil Vidyapeeth (Deemed-to-be-University), Pimpri, Pune, Maharashtra, India; c Faculty of Pharmaceutical Sciences, Graphic Era Hill University, Dehradun, India; d Centre for Promotion of Research, Graphic Era Deemed University, Dehradun, India; e Department of Pharmaceutics, Noida Institute of Engineering & Technology (Pharmacy Institute) Plot No.19, Knowledge Park-II, Greater Noida, India; f Saveetha Medical College and Hospital, Saveetha Institute of Medical and Technical Sciences, Saveetha University, Chennai, India; g Department of Anatomy, School of Medical Sciences and Research. Sharda University, Greater Noida, India

Dear editor,

We read with interest the recent article evaluating healthcare worker hand hygiene (HH) compliance before and after the COVID-19 pandemic and its relationship with alcohol-based hand rub (ABHR) consumption [1]. The study addresses an important question and has several methodological strengths, including a long observation period, use of the World Health Organization ‘Five Moments’ framework, and the combination of direct observation with consumption-based metrics, which aligns with current recommendations to triangulate process measures of HH performance [2,3].

At the same time, several aspects of the design and analysis warrant clarification to support robust interpretation. First, although the study is described as cross-sectional, HH compliance was assessed in two distinct periods (2018–2019 and 2022–2023), while ABHR consumption was tracked annually from 2018 to 2023. This corresponds more closely to repeated cross-sectional auditing paired with a time series of ABHR use. Labelling the design explicitly as such and analysing it accordingly (e.g. modelling time trends or period effects) would better reflect the underlying structure and avoid implying a single, static cross section [4].

Second, the decision to report HH compliance only for the prepandemic and postpandemic periods, while relying on ABHR consumption alone for the peak pandemic years, creates an asymmetry between behavioural and proxy measures. ABHR distribution from the warehouse is a pragmatic indicator but is known to be an imperfect surrogate for actual HH behaviour, particularly during crises when the product may be used outside clinical care or stockpiled [2,5]. This raises the possibility of ecological bias when inferring individual-level HH performance from aggregate consumption data [4]. A clearer statement of how ABHR data were cleaned and to what extent non-clinical use might have inflated estimates would strengthen confidence in the Doses of Hand Disinfection per 1000 Patient-Days (DMPPD) metric.

Relatedly, the calculation and interpretation of DMPPD could be reported with greater precision. Dividing ABHR consumption per 1000 patient-days by an assumed 3 mL per hand rub is consistent with prior work [2,5], but neither it is entirely clear whether inpatient-only or hospital-wide ABHR volumes were used in this denominator nor is the rationale for the 3-mL assumption discussed. Moreover, DMPPD is fundamentally an aggregate measure and should be interpreted as an average number of hand rub events per patient-day at the population level, rather than as the number of times an individual worker disinfects a single patient’s hands per day. Misinterpretation of such ecological averages is a well-described risk in health services research [4].

Finally, the authors acknowledge variation in observation opportunities and educational activities between the prepandemic and postpandemic periods. Unequal observation intensity and potential changes in staff awareness can introduce measurement and Hawthorne-related biases in directly observed HH compliance [3]. Formal statistical comparison of proportions (or regression models accounting for ward, profession, and period) could help quantify whether the apparent stability in overall compliance truly reflects unchanged behaviour or is partly an artefact of differing observation conditions.

This study makes a valuable contribution by spanning prepandemic and postpandemic periods and by combining observational and consumption data. Clarifying the underlying design, refining the reporting and interpretation of DMPPD, and more fully addressing the limitations of ABHR as a behavioural proxy would further enhance the methodological robustness and interpretability of the findings.

## CRediT authorship contribution statement

**S.N. Katkuri:** Conceptualization, Writing – original draft, Writing – review & editing. **V. Vadhithala:** Conceptualization, Writing – original draft, Writing – review & editing. **A. Kumar:** Writing – original draft, Writing – review & editing. **S. Verma:** Writing – original draft, Writing – review & editing. **D. Dedeepya:** Visualization, Writing – review & editing.

## Ethics statement

Not applicable.

## Funding sources

No funding was received for this study.

## Conflict of interest statement

None declared.
